# Supplementary material for: Single-defect spectroscopy in the shortwave infrared
Source: Nat Commun. 2019 Jun 17;10:2672. doi: 10.1038/s41467-019-10788-8 (PMC6572808; doi:10.1038/s41467-019-10788-8)
Supplement: Supplementary file 1 — Supplementary Information [file 41467_2019_10788_MOESM1_ESM.pdf]

Supplementary Information for

Single-Defect Spectroscopy in the Shortwave Infrared

Wu et al.

Index of Supplementary Figures

Supplementary Figure 1. Schematic for the photolithographic fabrication of the imaging substrate that contains fiduciary markers for sample drift correction in the shortwave infrared.

Supplementary Figure 2. The polystyrene/gold (PS/Au) substrate increased the PL intensity by ca. 5-fold.

Supplementary Figure 3. Proposed mechanism for drift correction in the shortwave infrared.

Supplementary Figure 4. Non-destructive readout significantly improves the signal-to-noise ratio in the shortwave IR.

Supplementary Figure 5. The normalized PL spectra of a (6,5)-SWCNT with two types of defects, methoxyaryl and nitroaryl.

Supplementary Figure 6. Incorporating two types of defects in the same nanotube.

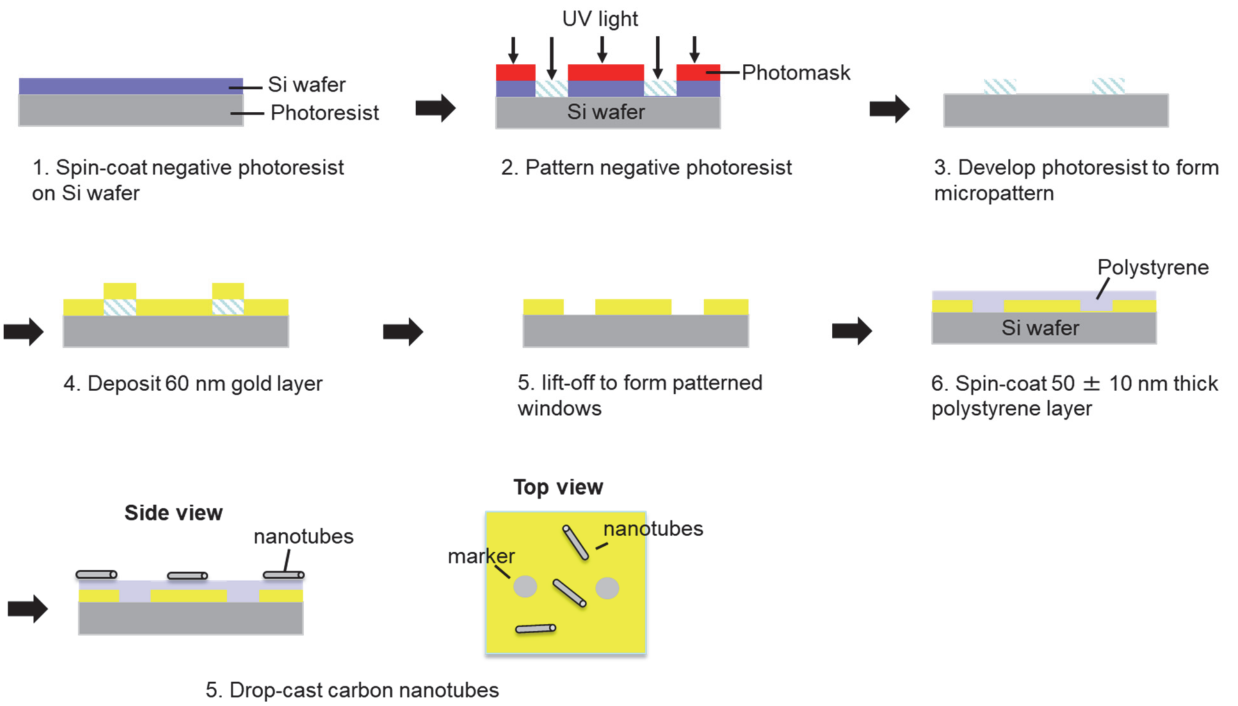

Supplementary Figure 1 | Schematic for photolithographic fabrication of the imaging substrate that contains fiduciary markers for sample drift correction in the shortwave infrared.

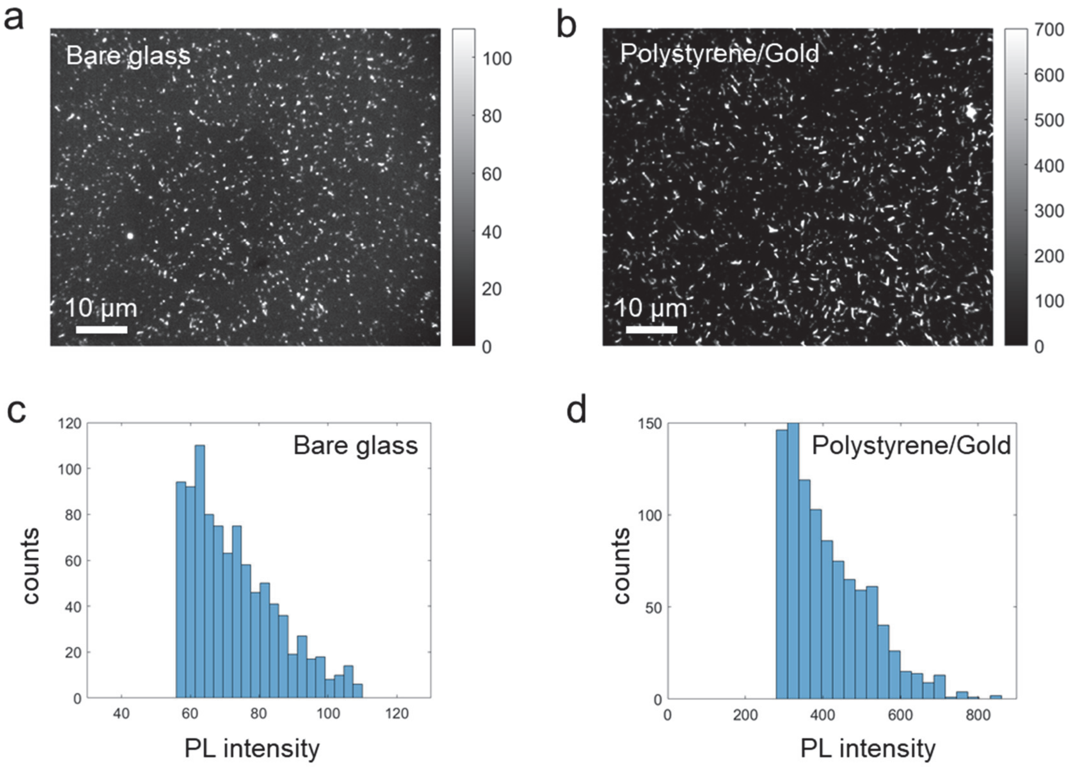

Supplementary Figure 2 | The polystyrene/gold (PS/Au) substrate increased the PL intensity by ca. 5-fold. PL images of SWCNTs on (a) bare glass and (b) the PS/Au substrate. The excitation wavelength was 730 nm and the integration time was 2 s. The color bar is the PL intensity ADU count. (c, d) The corresponding histograms of the SWCNT PL intensity distribution based on the PL images shown in (a) and (b).

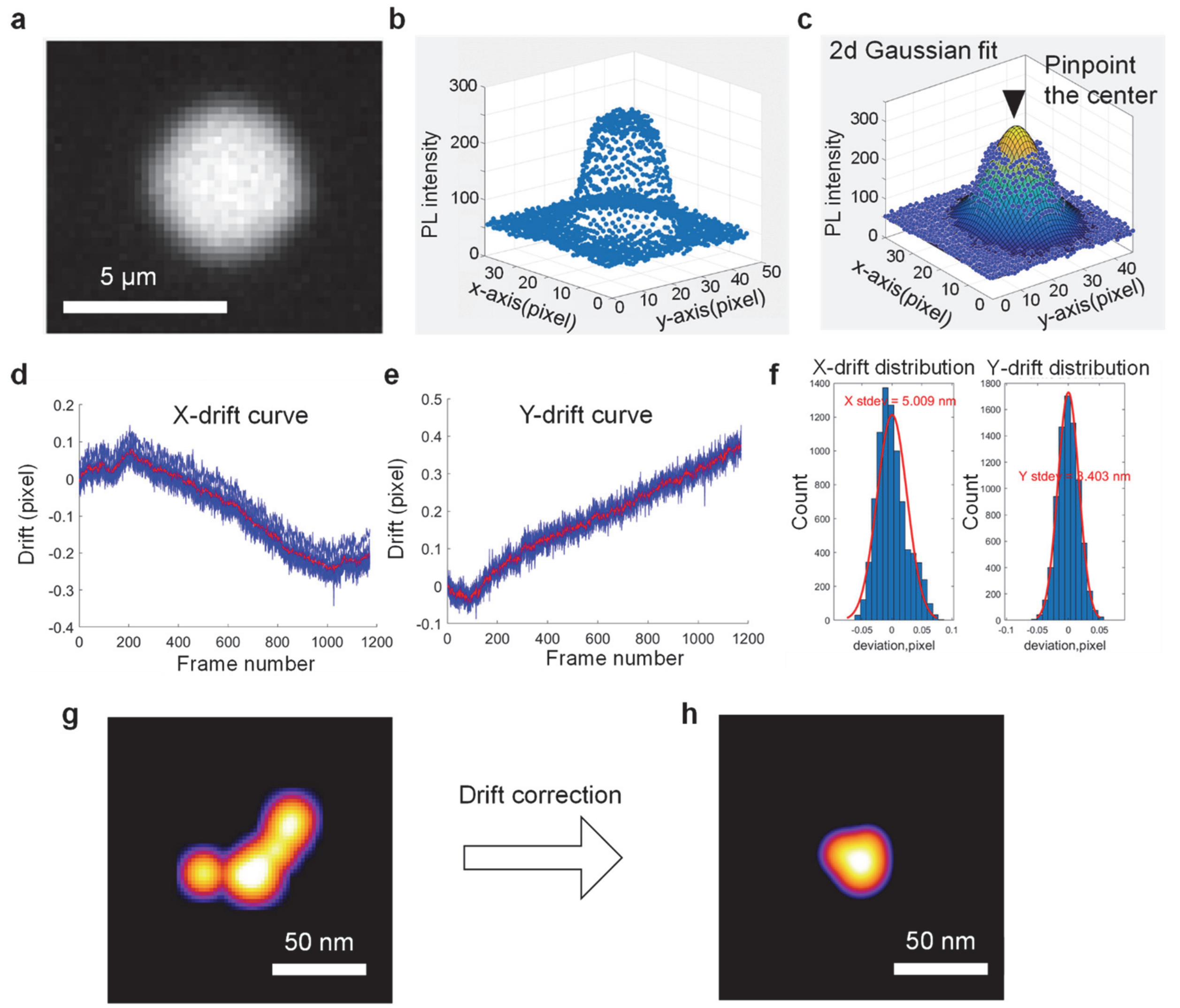

Supplementary Figure 3 | Proposed mechanism for drift correction in the shortwave infrared. **a**, The PL image, **b**, intensity profile, and **c**, center position of an individual fiduciary marker. Note the center position is located by fitting the intensity profile with a 2D Gaussian. This process was repeated for all frames in a time sequence, allowing us to plot the center positions versus time and construct a trajectory of the corresponding sample drift in the **d,** X and **e,** Y directions. At least five such trajectories (blue in d and e) from different markers in the same field of view were averaged together (red in d and e) and applied to correct the sub-pixel stage drift. **f**, The drift curve of an individual marker. The standard deviation from the averaged one defines the precision of this drift correction, which is determined to be 5 nm. Example of super-resolved defect locations **g,** before and **h,** after the drift correction using the obtained average X and Y sample drift trajectories.

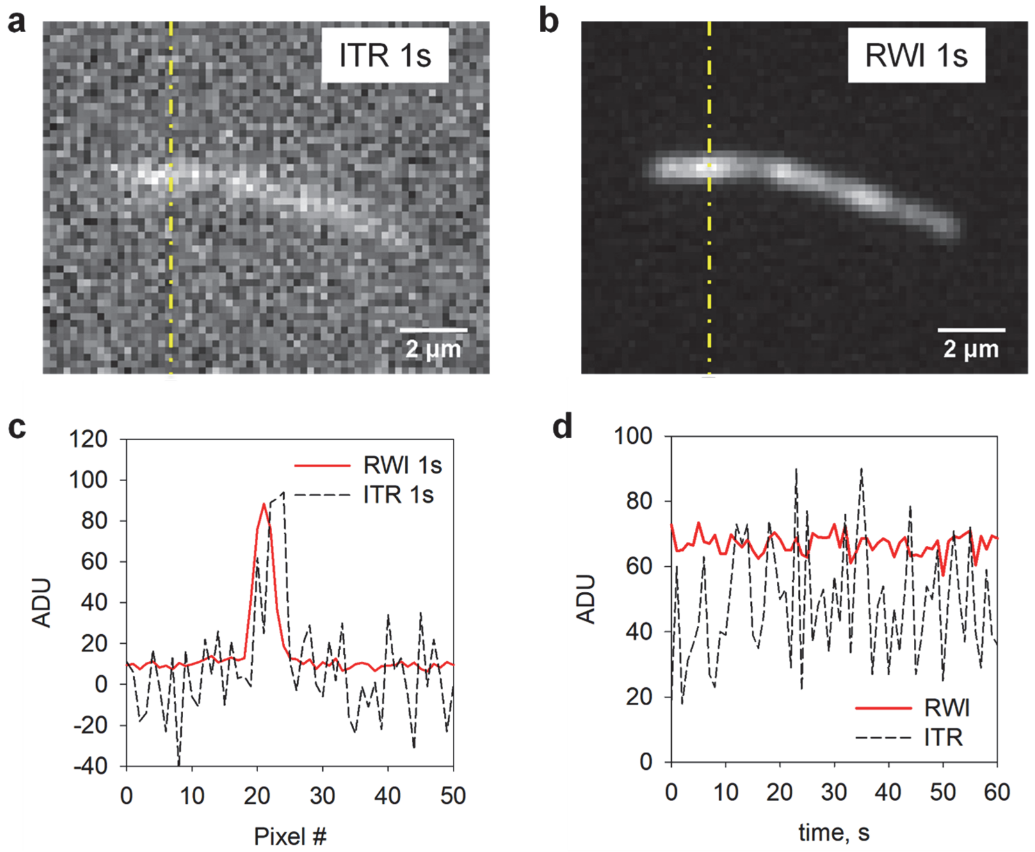

Supplementary Figure 4 | Non-destructive readout improves the signal-to-noise ratio in the shortwave IR. PL images of the same SWCNT taken in the **a,** ITR mode and the **b,** RWI mode with 1 s integration time. **c,** PL intensity profiles along the yellow line in (a) and (b) show much higher signal-to-noise ratio in the RWI mode (red) compared to the ITR mode (black). **d,** The PL time trajectory from the same pixel on the nanotube as shown in (a) and (b), which demonstrates nearly 8-times less fluctuation in intensity, with a standard deviation of 2.6 ADU (*vs.* 20.1 ADU for ITR).

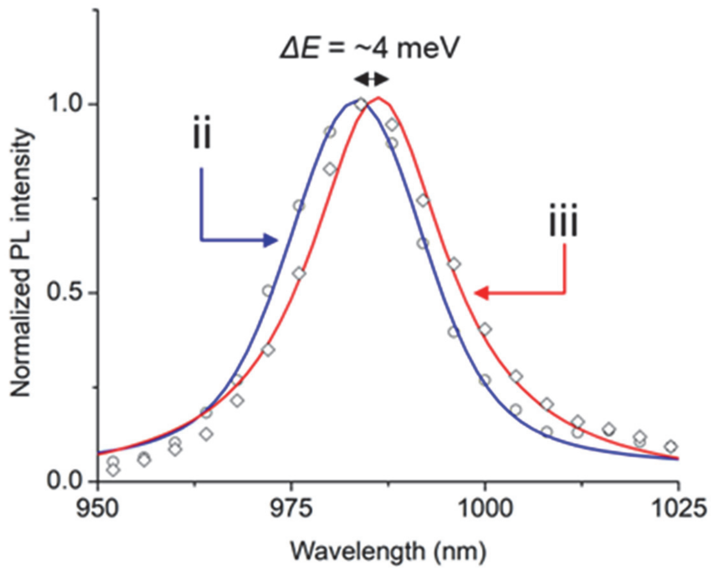

Supplementary Figure 5 | The normalized PL spectra of a (6,5)-SWCNT with two types of defects, methoxyaryl and nitroaryl. The E_{11} emission of at site iii has a 4 meV redshift from that of site ii.

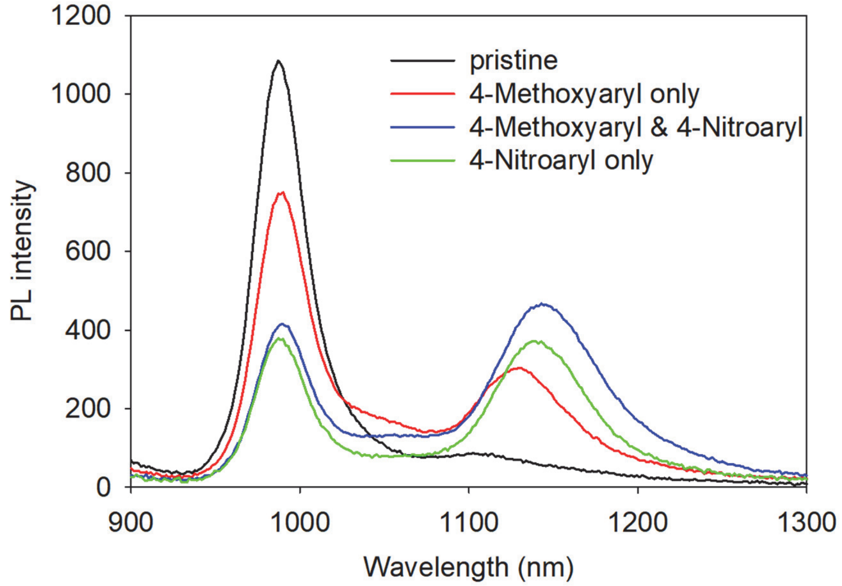

Supplementary Figure 6 | Incorporating two types of defects in the same nanotube. The ensemble level PL spectra of the pristine (6,5)-SWCNT sample (black), reacting first with 4-methxoyaryl diazonium (red), then with 4-nitroaryl diazonium (blue), and only with 4-nitroaryl diazonium (green). The excitation wavelength is 565 nm and the integration time is 3 s.
