# Supplementary material for: Single-defect spectroscopy in the shortwave infrared
Source: Nat Commun. 2019 Jun 17;10:2672. doi: 10.1038/s41467-019-10788-8 (PMC6572808; doi:10.1038/s41467-019-10788-8)
Supplement: Supplementary file 3 — Description of Additional Supplementary Files [file 41467_2019_10788_MOESM3_ESM.docx]

**Description of Supplementary Files**

**File Name:** **Supplementary Movie 1.**

**Description:** Photoluminescence blinking of a defect site in a nanotube semiconductor. The scale bar is 500 nm.
